# *N*-(5-Bromo­pyridin-2-yl)acetamide

**DOI:** 10.1107/S1600536811027553

**Published:** 2011-07-16

**Authors:** Hoong-Kun Fun, Tara Shahani, Rajesha Kumar, Arun M. Isloor, Kammasandra N. Shivananda

**Affiliations:** a X-ray Crystallography Unit, School of Physics, Universiti Sains Malaysia, 11800 USM, Penang, Malaysia; b Department of Chemistry, National Institute of Technology–Karnataka, Surathkal, Mangalore 575 025, India; c Schulich Faculty of Chemistry, Technion Israel Institute of Technology, Haifa 32000, Israel

## Abstract

The asymmetric unit of the title compound, C_7_H_7_BrN_2_O, contains two mol­ecules, in one of which the methyl H atoms are disorderd over two orientations in a 0.57 (3):0.43 (3) ratio. The dihedral angles between the pyridine rings and the acetamide groups are 7.27 (11) and 8.46 (11)°. In the crystal, mol­ecules are linked by N—H⋯O and C—H⋯O hydrogen bonds generating bifurcated *R*_2_^1^(5) ring motifs, which in turn lead to [110] chains.

## Related literature

For background to the acetyl­ation of amines, see: Greene & Wuts (1999 ▶); Moore *et al.* (1940 ▶); Suyama & Gerwick (2006 ▶). For a related structure, see: Loureiro *et al.* (2008 ▶). For further synthetic information, see: Augustine *et al.* (2011 ▶); Sollogoub *et al.* (2002 ▶).
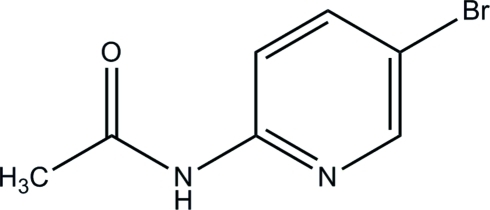


## Experimental

### 

#### Crystal data

C_7_H_7_BrN_2_O
*M* *_r_* = 215.06
Triclinic, 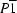

*a* = 4.0014 (3) Å
*b* = 8.7232 (6) Å
*c* = 23.0626 (18) Å
α = 82.127 (1)°
β = 86.897 (1)°
γ = 85.932 (1)°
*V* = 794.60 (10) Å^3^
*Z* = 4
Mo *K*α radiation
μ = 5.11 mm^−1^
*T* = 296 K
0.77 × 0.15 × 0.09 mm

#### Data collection

Bruker SMART APEXII CCD diffractometer
Absorption correction: multi-scan (*SADABS*; Bruker, 2009) ▶ *T*_min_ = 0.111, *T*_max_ = 0.665
13194 measured reflections
5134 independent reflections
3193 reflections with *I* > 2σ(*I*)
*R*_int_ = 0.025

#### Refinement

*R*[*F* ^2^ > 2σ(*F* ^2^)] = 0.031
*wR*(*F* ^2^) = 0.081
*S* = 1.00
5134 reflections
201 parameters
H-atom parameters constrained
Δρ_max_ = 0.35 e Å^−3^
Δρ_min_ = −0.25 e Å^−3^

### 

Data collection: *APEX2* (Bruker, 2009 ▶); cell refinement: *SAINT* (Bruker, 2009 ▶); data reduction: *SAINT*; program(s) used to solve structure: *SHELXTL* (Sheldrick, 2008 ▶); program(s) used to refine structure: *SHELXTL*; molecular graphics: *SHELXTL*; software used to prepare material for publication: *SHELXTL* and *PLATON* (Spek, 2009 ▶).

## Supplementary Material

Crystal structure: contains datablock(s) global, I. DOI: 10.1107/S1600536811027553/hb5933sup1.cif

Structure factors: contains datablock(s) I. DOI: 10.1107/S1600536811027553/hb5933Isup2.hkl

Supplementary material file. DOI: 10.1107/S1600536811027553/hb5933Isup3.cml

Additional supplementary materials:  crystallographic information; 3D view; checkCIF report

## Figures and Tables

**Table 1:** Hydrogen-bond geometry (Å, °)

*D*—H⋯*A*	*D*—H	H⋯*A*	*D*⋯*A*	*D*—H⋯*A*
N2*A*—H1*NA*⋯O1*B*^i^	0.85	2.16	3.001 (2)	169
N2*B*—H1*NB*⋯O1*A*^ii^	0.83	2.20	2.985 (2)	159
C7*A*—H7*AA*⋯O1*B*^i^	1.10	2.54	3.476 (3)	142

Symmetry codes: (i) 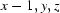; (ii) 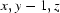.

**Fig. 1.**
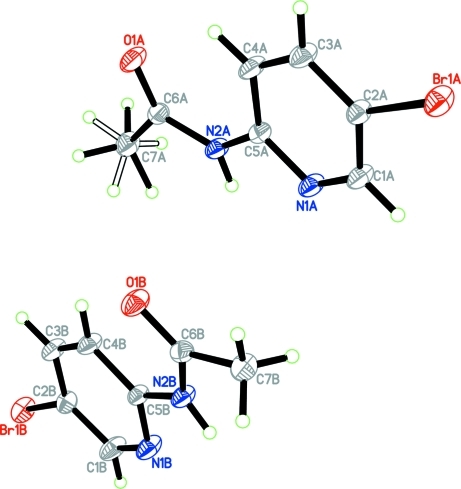
The molecular structure of the title compound, showing 20% probability displacement ellipsoids.

**Fig. 2.**
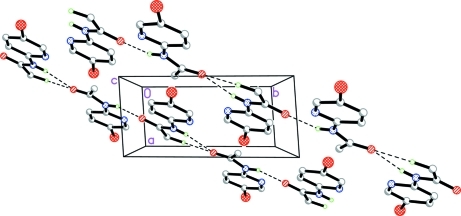
The crystal packing of the title compound, showing chains along the [110] direction. Only the major component is shown.

## supplementary crystallographic information

### Comment

The acetylation of amines is an important method for protection (Greene & Wuts, 1999) of this basic functionality that is an important part of many natural products and medicinally important compounds such as sulphanilamide (Moore *et al.*, 1940). In addition, certain natural products and medicinal compounds contain the acetamide functionality as part of the native compound or drug. Examples include epiquinamide, a compound isolated from a poison frog (Suyama *et al.*, 2006) and Tylenol a common analgesic compound. Prompted by these, we synthesized the title compound, (I), and determined its crystal structure.

The asymmetric unit of (I) consists of two independent molecules of *N*-(5-bromopyridin-2-yl)acetamide (A & B) as shown in Fig. 1. In molecule A, the methyl hydrogen atoms are disordered over two sets of sites, with occupancy ratio of 0.57 (3):0.43 (3). The pyridine (N1A/C1A–C5A)/(N1B/C1B–C5B) rings are essentially planar, with maximum deviations of 0.006 (2) Å for atom C4A and 0.004 (2) Å for atom N1B, respectively. The dihedral angle between the pyridine (N1A/C1A–C5A)/(N1B/C1B–C5B) rings and acetamide (N2A/O1A/C5A–C7A)/ (N2B/O1B/C5B–C7B) groups are 7.27 (11)° and 8.46 (11)° respectively. The bond lengths and angles are normal and comparable to those in a related structure (Loureiro *et al.*, 2008).

In the crystal (Fig. 2), the molecules are linked by intermolecular N2A—H1NA···O1B, N2B—H1NB···O1A and C7A—H7AA···O1B hydrogen bonds (Table 1) generating a bifurcated *R*^1^_2_(5) ring motif, resulting in supramolecular [1 1 0] chains.

### Experimental

(1*E*)-1-(5-Bromopyridin-2-yl)-*N*-hydroxyethanimine (2 g, 0.0093 mol) was taken in *N,N* dimethyl formamide (20 ml) at 25–26°C under a nitrogen atmosphere. Propylphosphonic anhydride (0.6 g, 0.00093 mol, 50% solution in ethylacetate) was added at the same temperature (Augustine *et al.*, 2011). The reaction mixture was heated to 100°C for 5 hrs. The reaction mixture was cooled to 25–26°C and quenched onto ice-cold water. The precipitated white solid was filtered and dried under vacuum to get the desired product as a white solid which was then recrystallized from ethanol (Sollogoub *et al.*, 2002) to yield colourless needles of (I). Yield 1.89 g (94.5%) *Mp*. 447–449 K.

### Refinement

All the H atoms were positioned geometrically [C–H = 0.9300 to 1.1046 Å, N–H = 0.8514 to 0.9600 Å] and were refined using a riding model, with *U*_iso_(H) =1.2 or 1.5*U*_iso_(C). One set of the methyl hydrogen atoms are disordered over two sets of sites, with occupancy ratio of 0.57 (3):0.43 (3).

### Crystal data

**Table:**

C_7_H_7_BrN_2_O	*Z* = 4
*M**_r_* = 215.06	*F*(000) = 424
Triclinic, *P*1	*D*_x_ = 1.798 Mg m^−^^3^
Hall symbol: -P 1	Mo *K*α radiation, λ = 0.71073 Å
*a* = 4.0014 (3) Å	Cell parameters from 3316 reflections
*b* = 8.7232 (6) Å	θ = 2.8–30.5°
*c* = 23.0626 (18) Å	µ = 5.11 mm^−^^1^
α = 82.127 (1)°	*T* = 296 K
β = 86.897 (1)°	Needle, colourless
γ = 85.932 (1)°	0.77 × 0.15 × 0.09 mm
*V* = 794.60 (10) Å^3^	

### Data collection

**Table:**

Bruker SMART APEXII CCD diffractometer	5134 independent reflections
Radiation source: fine-focus sealed tube	3193 reflections with *I* > 2σ(*I*)
graphite	*R*_int_ = 0.025
φ and ω scans	θ_max_ = 31.2°, θ_min_ = 1.8°
Absorption correction: multi-scan (*SADABS*; Bruker, 2009)	*h* = −5→5
*T*_min_ = 0.111, *T*_max_ = 0.665	*k* = −12→12
13194 measured reflections	*l* = −33→33

### Refinement

**Table:**

Refinement on *F*^2^	Primary atom site location: structure-invariant direct methods
Least-squares matrix: full	Secondary atom site location: difference Fourier map
*R*[*F*^2^ > 2σ(*F*^2^)] = 0.031	Hydrogen site location: inferred from neighbouring sites
*wR*(*F*^2^) = 0.081	H-atom parameters constrained
*S* = 1.00	*w* = 1/[σ^2^(*F*_o_^2^) + (0.036*P*)^2^ + 0.0264*P*] where *P* = (*F*_o_^2^ + 2*F*_c_^2^)/3
5134 reflections	(Δ/σ)_max_ = 0.006
201 parameters	Δρ_max_ = 0.35 e Å^−^^3^
0 restraints	Δρ_min_ = −0.25 e Å^−^^3^

### Special details

**Table:**

Geometry. All e.s.d.'s (except the e.s.d. in the dihedral angle between two l.s. planes) are estimated using the full covariance matrix. The cell e.s.d.'s are taken into account individually in the estimation of e.s.d.'s in distances, angles and torsion angles; correlations between e.s.d.'s in cell parameters are only used when they are defined by crystal symmetry. An approximate (isotropic) treatment of cell e.s.d.'s is used for estimating e.s.d.'s involving l.s. planes.
Refinement. Refinement of *F*^2^ against ALL reflections. The weighted *R*-factor *wR* and goodness of fit *S* are based on *F*^2^, conventional *R*-factors *R* are based on *F*, with *F* set to zero for negative *F*^2^. The threshold expression of *F*^2^ > σ(*F*^2^) is used only for calculating *R*-factors(gt) *etc*. and is not relevant to the choice of reflections for refinement. *R*-factors based on *F*^2^ are statistically about twice as large as those based on *F*, and *R*- factors based on ALL data will be even larger.

### Fractional atomic coordinates and isotropic or equivalent isotropic displacement parameters (Å^2^)

**Table:**

	*x*	*y*	*z*	*U*_iso_*/*U*_eq_	Occ. (<1)
Br1A	0.84865 (6)	0.73249 (2)	0.465121 (9)	0.05565 (9)	
O1A	0.4679 (4)	0.97838 (16)	0.73640 (6)	0.0613 (5)	
N1A	0.4235 (5)	0.63040 (18)	0.63081 (7)	0.0492 (4)	
N2A	0.3137 (4)	0.75247 (17)	0.71180 (6)	0.0433 (4)	
H1NA	0.2221	0.6674	0.7226	0.052*	
C1A	0.5431 (6)	0.6264 (2)	0.57599 (9)	0.0509 (5)	
H1AA	0.5303	0.5356	0.5595	0.061*	
C2A	0.6845 (5)	0.7499 (2)	0.54257 (8)	0.0433 (4)	
C3A	0.7046 (6)	0.8840 (2)	0.56632 (9)	0.0517 (5)	
H3AA	0.7967	0.9695	0.5444	0.062*	
C4A	0.5868 (6)	0.8907 (2)	0.62322 (9)	0.0515 (5)	
H4AA	0.6018	0.9801	0.6405	0.062*	
C5A	0.4449 (5)	0.7612 (2)	0.65427 (8)	0.0397 (4)	
C6A	0.3318 (5)	0.8566 (2)	0.74991 (8)	0.0427 (4)	
C7A	0.1744 (6)	0.8125 (3)	0.80974 (9)	0.0568 (6)	
H7AA	0.0849	0.6942	0.8167	0.085*	0.57 (3)
H7AB	−0.0154	0.9051	0.8188	0.085*	0.57 (3)
H7AC	0.3210	0.8117	0.8427	0.085*	0.57 (3)
H7AD	0.2003	0.8929	0.8334	0.085*	0.43 (3)
H7AE	0.2826	0.7172	0.8275	0.085*	0.43 (3)
H7AF	−0.0598	0.7992	0.8067	0.085*	0.43 (3)
Br1B	0.14559 (6)	0.24850 (3)	1.034407 (9)	0.05895 (9)	
O1B	0.9318 (4)	0.47747 (16)	0.76218 (6)	0.0586 (4)	
N1B	0.4457 (5)	0.13387 (19)	0.87208 (7)	0.0566 (5)	
N2B	0.6803 (4)	0.25146 (17)	0.78696 (6)	0.0464 (4)	
H1NB	0.6598	0.1634	0.7785	0.056*	
C1B	0.3245 (7)	0.1341 (2)	0.92681 (10)	0.0594 (6)	
H1BA	0.2365	0.0441	0.9461	0.071*	
C2B	0.3226 (5)	0.2599 (2)	0.95615 (8)	0.0443 (5)	
C3B	0.4515 (6)	0.3934 (2)	0.92795 (9)	0.0513 (5)	
H3BA	0.4549	0.4804	0.9471	0.062*	
C4B	0.5752 (6)	0.3965 (2)	0.87127 (9)	0.0506 (5)	
H4BA	0.6621	0.4857	0.8511	0.061*	
C5B	0.5681 (5)	0.2635 (2)	0.84446 (8)	0.0403 (4)	
C6B	0.8508 (5)	0.3553 (2)	0.74883 (8)	0.0430 (4)	
C7B	0.9337 (6)	0.3063 (2)	0.68947 (8)	0.0535 (5)	
H7BA	1.0711	0.3804	0.6667	0.080*	
H7BB	0.7302	0.3009	0.6698	0.080*	
H7BC	1.0528	0.2062	0.6939	0.080*	

### Atomic displacement parameters (Å^2^)

**Table:**

	*U*^11^	*U*^22^	*U*^33^	*U*^12^	*U*^13^	*U*^23^
Br1A	0.06525 (17)	0.05384 (14)	0.05063 (13)	−0.01839 (11)	0.01455 (10)	−0.01617 (9)
O1A	0.0935 (13)	0.0446 (8)	0.0504 (8)	−0.0284 (8)	0.0052 (8)	−0.0140 (6)
N1A	0.0661 (12)	0.0349 (8)	0.0487 (9)	−0.0178 (8)	0.0068 (8)	−0.0091 (7)
N2A	0.0546 (11)	0.0322 (8)	0.0439 (9)	−0.0118 (7)	0.0042 (8)	−0.0063 (6)
C1A	0.0665 (15)	0.0360 (9)	0.0532 (11)	−0.0158 (10)	0.0074 (10)	−0.0145 (8)
C2A	0.0470 (12)	0.0400 (10)	0.0445 (10)	−0.0104 (9)	0.0036 (9)	−0.0099 (8)
C3A	0.0658 (15)	0.0391 (10)	0.0520 (11)	−0.0220 (10)	0.0111 (10)	−0.0080 (8)
C4A	0.0726 (16)	0.0332 (9)	0.0519 (11)	−0.0193 (10)	0.0085 (10)	−0.0128 (8)
C5A	0.0407 (11)	0.0332 (9)	0.0463 (10)	−0.0064 (8)	−0.0009 (8)	−0.0074 (7)
C6A	0.0502 (12)	0.0378 (9)	0.0413 (9)	−0.0058 (9)	−0.0029 (8)	−0.0076 (7)
C7A	0.0728 (16)	0.0567 (12)	0.0427 (11)	−0.0150 (12)	0.0061 (10)	−0.0107 (9)
Br1B	0.06627 (17)	0.06192 (15)	0.05093 (13)	−0.02114 (12)	0.01604 (11)	−0.01425 (10)
O1B	0.0785 (11)	0.0464 (8)	0.0529 (8)	−0.0275 (8)	0.0091 (7)	−0.0069 (6)
N1B	0.0841 (14)	0.0414 (9)	0.0473 (9)	−0.0249 (9)	0.0121 (9)	−0.0120 (7)
N2B	0.0627 (12)	0.0340 (8)	0.0443 (9)	−0.0145 (8)	0.0051 (8)	−0.0087 (6)
C1B	0.0811 (17)	0.0427 (11)	0.0564 (12)	−0.0266 (11)	0.0164 (12)	−0.0103 (9)
C2B	0.0432 (12)	0.0468 (10)	0.0444 (10)	−0.0127 (9)	0.0056 (8)	−0.0088 (8)
C3B	0.0667 (15)	0.0384 (10)	0.0517 (11)	−0.0149 (10)	0.0071 (10)	−0.0141 (8)
C4B	0.0701 (15)	0.0328 (9)	0.0500 (11)	−0.0175 (9)	0.0070 (10)	−0.0063 (8)
C5B	0.0452 (12)	0.0333 (9)	0.0438 (10)	−0.0093 (8)	−0.0007 (8)	−0.0073 (7)
C6B	0.0466 (12)	0.0375 (9)	0.0448 (10)	−0.0068 (9)	−0.0016 (8)	−0.0031 (8)
C7B	0.0610 (15)	0.0532 (12)	0.0471 (11)	−0.0127 (11)	0.0065 (10)	−0.0081 (9)

### Geometric parameters (Å, °)

**Table:**

Br1A—C2A	1.8914 (18)	C7A—H7AF	0.9600
O1A—C6A	1.223 (2)	Br1B—C2B	1.8951 (18)
N1A—C1A	1.331 (3)	O1B—C6B	1.218 (2)
N1A—C5A	1.338 (2)	N1B—C1B	1.328 (3)
N2A—C6A	1.356 (2)	N1B—C5B	1.331 (2)
N2A—C5A	1.395 (2)	N2B—C6B	1.365 (2)
N2A—H1NA	0.8514	N2B—C5B	1.392 (2)
C1A—C2A	1.374 (3)	N2B—H1NB	0.8288
C1A—H1AA	0.9300	C1B—C2B	1.365 (3)
C2A—C3A	1.367 (3)	C1B—H1BA	0.9300
C3A—C4A	1.378 (3)	C2B—C3B	1.373 (3)
C3A—H3AA	0.9300	C3B—C4B	1.370 (3)
C4A—C5A	1.391 (3)	C3B—H3BA	0.9300
C4A—H4AA	0.9300	C4B—C5B	1.390 (3)
C6A—C7A	1.498 (3)	C4B—H4BA	0.9300
C7A—H7AA	1.1046	C6B—C7B	1.503 (3)
C7A—H7AB	1.1020	C7B—H7BA	0.9600
C7A—H7AC	0.9834	C7B—H7BB	0.9600
C7A—H7AD	0.9601	C7B—H7BC	0.9600
C7A—H7AE	0.9601		
			
C1A—N1A—C5A	117.99 (17)	H7AD—C7A—H7AE	109.5
C6A—N2A—C5A	127.87 (16)	C6A—C7A—H7AF	109.7
C6A—N2A—H1NA	120.4	H7AA—C7A—H7AF	60.9
C5A—N2A—H1NA	111.7	H7AB—C7A—H7AF	59.5
N1A—C1A—C2A	123.21 (18)	H7AC—C7A—H7AF	134.3
N1A—C1A—H1AA	118.4	H7AD—C7A—H7AF	109.5
C2A—C1A—H1AA	118.4	H7AE—C7A—H7AF	109.5
C3A—C2A—C1A	118.88 (18)	C1B—N1B—C5B	118.10 (17)
C3A—C2A—Br1A	121.10 (14)	C6B—N2B—C5B	128.33 (16)
C1A—C2A—Br1A	120.01 (14)	C6B—N2B—H1NB	119.6
C2A—C3A—C4A	119.17 (18)	C5B—N2B—H1NB	111.5
C2A—C3A—H3AA	120.4	N1B—C1B—C2B	123.26 (19)
C4A—C3A—H3AA	120.4	N1B—C1B—H1BA	118.4
C3A—C4A—C5A	118.69 (17)	C2B—C1B—H1BA	118.4
C3A—C4A—H4AA	120.7	C1B—C2B—C3B	118.78 (18)
C5A—C4A—H4AA	120.7	C1B—C2B—Br1B	120.11 (15)
N1A—C5A—C4A	122.06 (18)	C3B—C2B—Br1B	121.12 (15)
N1A—C5A—N2A	113.20 (16)	C4B—C3B—C2B	119.06 (18)
C4A—C5A—N2A	124.74 (16)	C4B—C3B—H3BA	120.5
O1A—C6A—N2A	122.27 (17)	C2B—C3B—H3BA	120.5
O1A—C6A—C7A	122.17 (17)	C3B—C4B—C5B	118.65 (18)
N2A—C6A—C7A	115.57 (17)	C3B—C4B—H4BA	120.7
C6A—C7A—H7AA	113.6	C5B—C4B—H4BA	120.7
C6A—C7A—H7AB	108.3	N1B—C5B—C4B	122.15 (18)
H7AA—C7A—H7AB	115.1	N1B—C5B—N2B	113.26 (16)
C6A—C7A—H7AC	115.8	C4B—C5B—N2B	124.59 (17)
H7AA—C7A—H7AC	102.8	O1B—C6B—N2B	122.50 (17)
H7AB—C7A—H7AC	100.6	O1B—C6B—C7B	122.78 (17)
C6A—C7A—H7AD	109.4	N2B—C6B—C7B	114.71 (16)
H7AA—C7A—H7AD	136.6	C6B—C7B—H7BA	109.5
H7AB—C7A—H7AD	53.5	C6B—C7B—H7BB	109.5
H7AC—C7A—H7AD	51.1	H7BA—C7B—H7BB	109.5
C6A—C7A—H7AE	109.4	C6B—C7B—H7BC	109.5
H7AA—C7A—H7AE	50.3	H7BA—C7B—H7BC	109.5
H7AB—C7A—H7AE	142.2	H7BB—C7B—H7BC	109.5
H7AC—C7A—H7AE	59.4		
			
C5A—N1A—C1A—C2A	−0.4 (4)	C5B—N1B—C1B—C2B	−0.7 (4)
N1A—C1A—C2A—C3A	0.0 (4)	N1B—C1B—C2B—C3B	0.1 (4)
N1A—C1A—C2A—Br1A	179.81 (18)	N1B—C1B—C2B—Br1B	−179.7 (2)
C1A—C2A—C3A—C4A	0.8 (4)	C1B—C2B—C3B—C4B	0.5 (4)
Br1A—C2A—C3A—C4A	−178.97 (18)	Br1B—C2B—C3B—C4B	−179.66 (18)
C2A—C3A—C4A—C5A	−1.2 (4)	C2B—C3B—C4B—C5B	−0.5 (4)
C1A—N1A—C5A—C4A	0.0 (3)	C1B—N1B—C5B—C4B	0.7 (4)
C1A—N1A—C5A—N2A	−179.75 (19)	C1B—N1B—C5B—N2B	−178.4 (2)
C3A—C4A—C5A—N1A	0.8 (3)	C3B—C4B—C5B—N1B	−0.1 (4)
C3A—C4A—C5A—N2A	−179.5 (2)	C3B—C4B—C5B—N2B	179.0 (2)
C6A—N2A—C5A—N1A	171.58 (19)	C6B—N2B—C5B—N1B	−172.3 (2)
C6A—N2A—C5A—C4A	−8.2 (3)	C6B—N2B—C5B—C4B	8.6 (4)
C5A—N2A—C6A—O1A	1.7 (3)	C5B—N2B—C6B—O1B	0.9 (3)
C5A—N2A—C6A—C7A	−178.2 (2)	C5B—N2B—C6B—C7B	−179.8 (2)

### Hydrogen-bond geometry (Å, °)

**Table:**

*D*—H···*A*	*D*—H	H···*A*	*D*···*A*	*D*—H···*A*
N2A—H1NA···O1B^i^	0.85	2.16	3.001 (2)	169
N2B—H1NB···O1A^ii^	0.83	2.20	2.985 (2)	159
C7A—H7AA···O1B^i^	1.10	2.54	3.476 (3)	142

Symmetry codes: (i) *x*−1, *y*, *z*; (ii) *x*, *y*−1, *z*.
